# Predictive factors for lymph node metastasis in papillary thyroid cancer patients undergoing neck dissection: insights from a large cohort study

**DOI:** 10.3389/fonc.2024.1447903

**Published:** 2024-10-25

**Authors:** Shuping Wu, Yu Liu, Xianhui Ruan, Xiangqian Zheng

**Affiliations:** ^1^ Department of Thyroid and Neck Tumor, Tianjin Medical University Cancer Institute and Hospital, National Clinical Research Center for Cancer, Tianjin's Clinical Research Center for Cancer, Key Laboratory of Cancer Prevention and Therapy, Tianjin, China; ^2^ Department of Head and Neck Surgery, Clinical Oncology School of Fujian Medical University & Fujian Cancer Hospital, Fuzhou, Fujian, China

**Keywords:** risk factors, neck dissection, lymph node, metastasis, thyroid cancer

## Abstract

**Background:**

This study aimed to investigate the risk factors and metastatic patterns in papillary thyroid cancer (PTC) patients undergoing lymph node dissection, offering guidance for clinical practice.

**Methods:**

A total of 924 PTC patients who underwent thyroidectomy with central neck dissection (CND) or lateral neck dissection (LND) between January 2021 and November 2022 were included in the analysis. The study investigated the relationships between clinicopathological characteristics, lymph node metastasis, and various risk factor.

**Results:**

Among the 924 PTC patients, the cervical lymph node metastasis rate was 59.1% (546 patients). Of these patients, 381 had central neck metastasis (CNM, 41.2%), while the remaining 165 patients had lateral neck metastasis (LNM, 17.9%). Factors associated with increased risk of CNM and LNM included larger tumor diameter, presence of multiple tumors, and capsular invasion (p<0.05). Male sex, age <55 years, larger tumor diameter (>0.85 cm), multiple tumors, capsular invasion, and absence of Hashimoto’s disease were identified as independent risk factors for CNM (p<0.05), with an AUC value of 0.722. CNM, maximum diameter >1.15 cm, and multiple tumors were independent risk factors for LNM (p<0.05), with an AUC of 0.699.

**Conclusion:**

These findings suggest that tailored neck dissection based on individual risk factors is crucial, particularly in cases of suspected LNM with larger tumors, CNM, multiple tumors, and capsular invasion.

## 1 Introduction

Thyroid cancer (TC) counts as one of the most common malignancies and the most common endocrine tumor (1). Although the mortality rate is low compared to other cancers, published studies have reported that the incidence of TC continues to increase in almost every country and region of the world. According to the latest statistics from the American Cancer Society and the National Cancer Institute, TC has become the third most prevalent malignancy in women, with a total cases toll of 823,800 in the United States (2). Data released by the China National Cancer Centre show that thyroid morbidity thyroid among all malignancies, and that they are increasing year after year, along with changes in lifestyle (3). Thyroid cancer has become an important disease affecting public health in both Eastern and Western countries, and surgery is the most common approach to treat thyroid cancer.

Papillary thyroid carcinoma(PTC), is the most prevalent type of thyroid cancer, representing 80-85% of all cases (4). Despite being considered an indolent tumor, PTC can metastasize to the lymph nodes surrounding the thyroid gland. Lymph node metastasis occurs frequently in approximately 40–50% of PTC patients (5, 6). This typically involves central neck metastasis (CNM) as well as lateral neck metastasis (LNM) (7). Research shows that pN1 patients suffer a higher rate of recurrence both among adults and children, and the N1b stage is an additional risk factor for the outcome of the disease (8–10). However, there is still controversy over the timing of surgery and the extent of lymph node dissection (10–13).

In this study, we aimed to explore the risk factors and metastatic trends of papillary thyroid cancer patients and provide advice for lymph node dissection. By retrospectively analyzing the clinicopathologic data of 924 consecutive PTC patients who underwent thyroidectomy with therapeutic lymph node dissection, we investigated various risk factors for different levels of lymphatic metastasis to provide advice on the extent of dissection.

## 2 Methods

### 2.1 Patients

A retrospective review involving all PTC patients in a single treatment group at the Department of Thyroid and Neck Tumor, Tianjin Medical University Cancer Institute and Hospital, between January 2022 and November 2023 was conducted. The inclusion criteria were as follows: 1) underwent thyroidectomy combined with unilateral or bilateral CND or modified radical neck dissection; 2) preoperative biopsy confirmed by pathology thyroid nodules are malignant; 3) patients with high suspicion of lateral lymph nodes on ultrasonography undergo lateral lymph node dissection (LLND), and the diagnosis is mainly confirmed by preoperative FNA measurement and thyroglobulin flushing.The exclusion criteria are as follows: 1) previous history of thyroid-associated surgery; 2) postoperative pathology combined with other thyroid cancers (medullary carcinoma, anaplastic carcinoma or poorly differentiated carcinoma); 3) patients’ clinical data are complete; 4) preoperative biopsy confirmed by pathology Thyroid nodules are malignant.

A total of 924 patients (679 females and 245 males) with an average age of 45.26±11.075 years were included in the study. Clinicopathologic features, such as sex, age at diagnosis, maximum diameter of the tumor, number of tumors, degree of capsular invasion, incidence of Hashimoto’s disease, number of lymph nodes dissected, and number of metastatic lymph nodes that were collected.The maximum tumour size measured macroscopically in one specimen slice. Histological examination reveals distinct areas of capsular invasion, where tumor cells exhibit a pattern of tunneling outward through the fibrous tumor capsule, creating either a bulbous or hook-shaped extension that extends beyond the outer edge of the capsule (11).

### 2.2 Thyroidectomy

Thyroid surgery was performed by a single treatment group to ensure that the surgical strategies were the same. All patients underwent intraoperative frozen sectioning to determine nodule malignancy. Therapeutic CND or LND was chosen based on preoperative examinations (including ultrasound, fine needle aspiration, wash-out of Tg from the suspicious lymph node, and CT or MRI), intraoperative exploration, and tumor characteristics. The patients all underwent a consistent dissection range(CND or LND).Central lymph nodes refer to level VI, the lateral lymph nodes included levels II, III, IV, and V. Informed consent for surgery was obtained preoperatively.

### 2.3 Statistical analysis

The data are expressed as the number (%) and M(P25, P75). The Mann‒Whitney test, χ2 test or Fisher’s exact test was used to analyze categorical variables, and multivariate logistic regression analysis was used to identify independent risk factors. Moreover, a receiver operating characteristic (ROC) curve based on the predicted logistic regression values was generated to determine the reference values of the identified risk factors. A p value of <0.05 was considered to indicate statistical significance. All the statistical analyses were performed with IBM SPSS statistics v24.0. software.

## 3 Results

### 3.1 Trends in metastasis among patients

We examined the characteristics of the 924 patients (245 females) enrolled in the study. A total of 59.1% (546 of 924 patients) of patients had lymph node metastasis, 381 (41.2%) had CNM only, and 165 (17.9%) had both CNM and LNM, as shown in **Table 1**.

**Table 1:** Patient characteristics.

Characteristics	n (%)
Gender	
Male	245 (26.5)
Female	679 (73.5)
Age at diagnosis	
<55	725 (78.5)
≥55	199 (21.5)
T stage	
pT1	843 (91.2)
pT2	51 (5.5)
pT3	11 (1.2)
pT4	19 (2.1)
N stage	
N1a	381 (41.2)
N1b	165 (17.9)

### 3.2 Comparison of clinicopathologic features for CNM risk of patients

Variable clinicopathologic features were included in the multivariate model to identify risk factors for CNM and LNM. The Mann‒Whitney test showed that the maximum diameter in the CNM group was significantly greater than that in the nonmetastasis group. Fisher’s exact test and the χ2 test revealed that males, patients aged <55 years, patients with multiple tumors, patients with capsular invasion, and patients without Hashimoto’s disease had a greater percentage of CNM (p<0.05), as shown in **Table 2**.

**Table 2:** Comparison of clinicopathologic features for CNM risk of patients.

Category	Number (%)	Central neck metastasis (number (%)/M(P25, P75))		χ^2/Z^	p-value
		CNM	Non-CNM		
Gender				9.094	0.003
Male	245 (26.5)	153 (62.4)	92 (37.6)		
Female	679 (73.5)	348 (51.3)	331 (48.7)		
Age at diagnosis				7.369	0.007
<55	725 (78.5)	410 (56.6)	315 (43.4)		
≥55	199 (21.5)	91 (45.7)	108 (54.3)		
Tumor maximum diameter (cm)		0.9 (0.6,1.5)	0.6 (0.4,0.8)	9.74	<0.001
Multiple tumors				28.869	<0.001
No	562 (60.8)	265 (47.2)	297 (52.8)		
Yes	362 (39.2)	236 (65.2)	126 (34.8)		
Capsular invasion				39.508	<0.001
No	542 (58.7)	247 (45.6)	295 (54.4)		
Yes	382 (41.3)	254 (66.5)	128 (33.5)		
Hashimoto’s disease				7.073	0.008
No	799 (86.5)	447 (55.9)	352 (44.1)		
yes	125 (13.5)	54 (43.2)	71 (56.8)		

**Figure 1:**
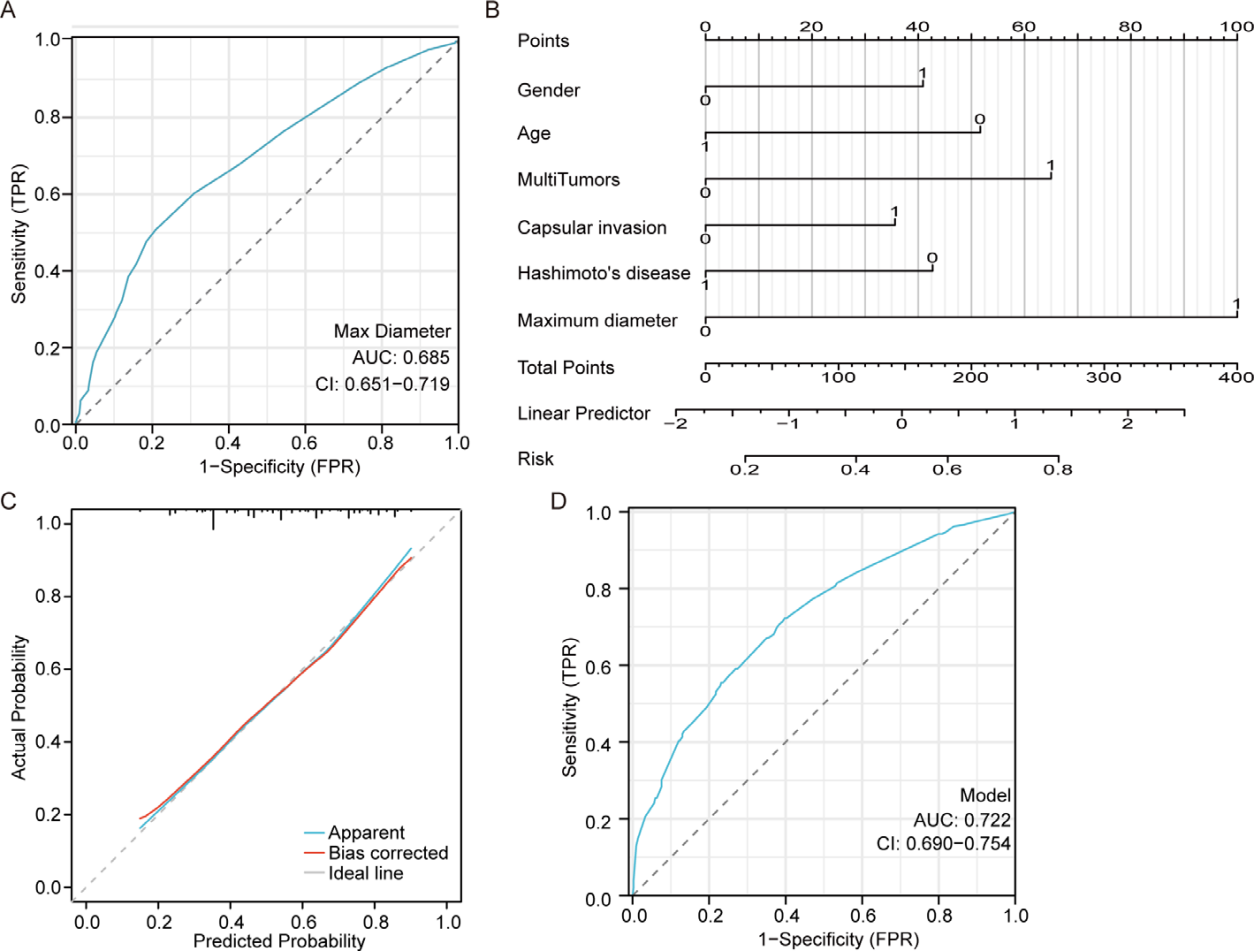
Central neck metastasis predictive model. **(A)** the ROC curve of maximum tumor diameter for predicting CNM. **(B)** a nomogram for predicting CNM. **(C)** the calibration curve for the nomogram. **(D)** the ROC curve of the nomogram for predicting CNM.


Moreover, the ROC curve based on the predicted CNM values suggested that the maximum tumor diameter had a referential meaning, with an AUC of 0. 685 cm and a cutoff value of 0.85 cm, as shown in **Figure 1A**. Multivariate logistic regression analysis indicated that male sex, age<55 years, larger maximum diameter (>0.85 cm), multiple tumors, capsular invasion, and the absence of Hashimoto’s disease were independent risk factors for CNM, as shown in **Figure 1B**. The equation for estimating CNM risk and the values of the predictors, obtained from the logistic regression coefficients, are given in the following equations:

$$\text{Prob}\{\text{CNM}=1\}=\frac{1}{1+\text{exp}(-\text{X}\;\text{β})}$$

, where


X β=−0.63−0.505∗Hashimoto's disease +0.411∗capsular invasion+0.764 ∗ multiple tumors−0.608∗age+0.474∗gender+1.2 ∗ maximum diameter

Hashimoto’s disease, capsular invasion, multiple tumors, maximum diameter >0.85cm, age>55, male=1, 0 otherwise.

The close alignment of the calibration curve to the 45° line in **Figure 1C** indicates strong validation on an absolute probability scale. The ROC curve based on the predicted values of the CNM suggested that the above independent risk factors had a referential meaning, with an AUC of 0.722 (95% CI 0.690-0.754) and a cutoff probability value of 0.487, as shown in **Figure 1D**.

### 3.3 Comparison of clinicopathologic features for LNM risk of patients

Apart from the analysis of CNM, we found the following risk factors for LNM: CNM, larger tumor of maximum diameter, multiple tumors, and capsular invasion (p<0.05), as shown in **Table 3**.

**Table 3:** Comparison of clinicopathologic features for LNM risk of patients.

Category	Number (%)	Lateral neck metastasis (number (%)/M(P25, P75))		χ^2/Z^	p-value
		LLNM	Non-LLNM		
Gender				0.116	0.733
Male	245 (26.5)	42 (17.1)	203 (82.9)		
Female	679 (73.5)	123 (18.1)	556 (81.9)		
Age at diagnosis				0.870	0.351
<55	725 (78.5)	125 (17.2)	600 (82.8)		
≥55	199 (21.5)	40 (20.1)	159 (79.9)		
Tumor maximum diameter (cm)		1.0 (0.6,1.5)	0.7 (0.5,1.1)	4.832	<0.001
Multiple tumors				40.928	<0.001
No	562 (60.8)	64 (11.4)	498 (88.6)		
Yes	362 (39.2)	101 (27.9)	261 (72.1)		
Capsular invasion				14.440	<0.001
No	542 (58.7)	75 (13.8)	467 (86.2)		
Yes	382 (41.3)	90 (23.6)	292 (76.4)		
Hashimoto’s disease				0.029	0.865
No	799 (86.5)	142 (17.8)	657 (82.2)		
yes	125 (13.5)	23 (18.4)	102 (81.6)		
CNM				22.54	<0.001
No	423 (45.8)	48 (11.3)	375 (88.7)		
yes	501 (54.2)	117 (23.4)	384 (76.6)		

**Figure 2:**
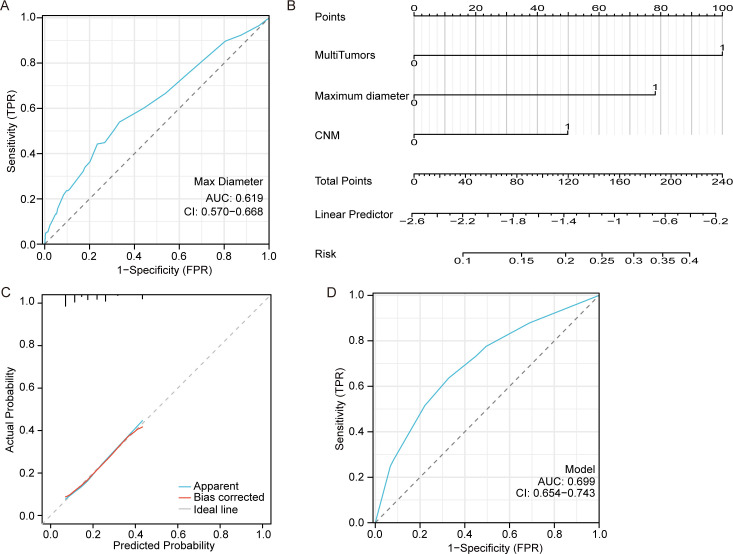
Model for predicting lateral neck metastasis. **(A)** The ROC curve of the maximum tumor diameter for predicting LNM. **(B)** A nomogram for predicting LNM. **(C)** The calibration curve for the nomogram. **(D)** The ROC curve of the nomogram for predicting LNM.


The ROC curve based on the predicted values of LNM suggested that the tumor maximum diameter had a reference meaning, with an AUC of 0.619 and a cutoff value of 1.15 cm, as shown in **Figure 2A**. Logistic regression analysis identified CNM, a maximum diameter >1.15 cm and multiple tumors as independent risk factors, as shown in **Figure 2B**. The equation for estimating LNM risk and the values of the predictors, obtained from the logistic regression coefficients, are given in the following equations:

$$\text{Prob}\{\text{LNM}=1\}=\frac{1}{1+\text{exp}(-\text{X}\;\text{β})}$$

, where


$$\begin{array}{l} \text{X}\;\text{β}=-2.584+0.506*\text{CNM}+1.015*\text{multiple}\;\text{tumors}+0.794\\ \begin{array}{c} \;*\; \end{array}\text{maximum}\;\text{diameter} \end{array}$$

Multiple tumors had a maximum diameter >1.15 cm, CNM=1, and 0 otherwise.

The calibration curve demonstrates a strong correlation between the predicted and observed values as shown in **Figure 2C**. Additionally, the ROC curve exhibits excellent discrimination with an AUC of 0.699 (95%CI 0.654-0.743) for predicting LNM and a cutoff Prob value of 0.194 as shown in **Figure 2D**.

## 4 Discussion

The treatment of thyroid cancer has been a subject of controversy for many years, particularly for patients with papillary thyroid carcinoma (PTC) who have early-stage, low-risk, or microcarcinoma disease, due to the generally favorable prognosis of most types of thyroid cancer. Whether patients should undergo surgery, thermal ablation, or placement under active surveillance remains controversial (12–16). Prophylactic lymphadenectomy is not recommended by the ATA guidelines for thyroid nodules and differentiated thyroid cancer because the procedure does not increase the survival rate over noninvasive treatments among T1-2 and cN0 differentiated thyroid cancer patients (17). However, the consensual opinion of Chinese experts, in cases of papillary thyroid microcarcinoma, is to advise prophylactic central neck lymph dissection as a Grade B recommendation because its efficacy has been proven with convincing evidence (18). The efficacy of a patient’s treatment should not solely rely on survival rates, but should also consider factors such as quality of life, emotional impact, and financial burden of a second surgery. These elements are crucial for the overall wellbeing of both the patient and their family. To avoid the damage caused by a second surgery, ensuring an adequate surgical extent in primary treatment is necessary. Due to improvements in surgical instruments and detailed preoperative evaluations (19, 20), complications from thyroid surgery are also decreasing. Complications caused by thyroidectomy and lymphadenectomy are mostly temporary and can be restricted within acceptable limits by skilled surgeons (21–23). Since the decision of surgery was made, identifying risk factors for lymph node metastasis can help determine the necessity of lymph node dissection and reduce the chance of a second surgery at the initial stage of treatment, thus improving quality of life.

Statistics show that the morbidity of PTC is greater among females than among males (24). Among the 924 patients included in our study, 679 were females. However, 62.4% of the male patients had CNM, which was significantly greater than that of the female patients (51.3%, p<0.05), which shows that men are at greater risk of lymph node metastasis, which agrees with similar past findings (25–27). Skip metastasis was occurred in 48 (11.3%) of these patients, and the probability of this phenomenon in previous studies ranged from 0.6% -22.5% (28, 29). Tumor diameter ≤10 mm, absence of BRAFV600E mutation, upper location of tumor were risk factors for skip metastasis in PTC patients (30, 31).

Due to the rising emphasis on disease prevention, there has been an increase in screening for thyroid-related diseases. As a result, the majority of thyroid cancer patients included in this study were diagnosed at an early stage of the disease. According to the analysis of the clinicopathologic features mentioned above, patients with papillary thyroid microcarcinoma (PTMC) had a lower metastatic rate in both the N1a and N1b stages, which indicates that T1a patients have a better illness status and a lower rate of recurrence. However, CNM was still found in up to 45% of these patients, which implies that almost half of PTMC patients may suffer from recurrence of lymph node metastasis if lymphadenectomy is insufficient or inappropriate, especially because ultrasound or CT has relatively low accuracy in determining metastatic central neck lymph nodes compared with metastatic lateral lymph nodes (32, 33).

However, a larger tumor represents a greater risk than a larger microcarcinoma. CNM and LNM occurred more often among patients whose maximum tumor diameter was larger than 0.85 cm or 1.15 cm, indicating that more attention should be given to patients who are beyond the T1 stage while performing lymphadenectomy to ensure that an adequate section is removed.

The greater probability of the occurrence of lymph node metastasis among younger patients has been reported by many researchers (27, 34, 35), and our study confirms this finding. Therefore, age at diagnosis less than 55 years was an independent risk factor for CNM patients (p<0.05). Capsular invasion plays an important role in the assessment of disease progression and recurrence (25–27, 36). We found that capsular invasion occurred in 66.5% of patients enrolled with CNM. ATA guidelines suggest CND for patients whose tumor diameter is larger than 4 cm or who show capsular invasion (17), which emphasizes the guiding significance of these indications. Multifocality is common in PTC (37, 38). Among the enrolled patients, 362 (39.2%) had multiple tumors according to pathological analysis, and 65.2% had CNM. Therefore, to ensure the completeness of the operation, complete resection of the thyroid lobe and standard dissection of the lymph nodes are needed during treatment. The above results indicate that in male patients who are younger than 55 years, whose tumor diameter is >0.85 cm, have multiple tumors and capsular invasion, and have no Hashimoto’s disease, more attention should be given when performing central neck lymphadenectomy. The AUC value of 0.722 confirms this significance or the above indications.

Among patients with LNM, only tumor size (>1.15 cm), capsular invasion, multiple tumors and CNM were risk factors for metastasis. This might be due to the advanced stage of PTC; the state of disease is dependent mainly on the features of the tumor and progression but not on patient characteristics such as sex or age. Logistic regression confirmed CNM, a maximum tumor diameter greater than 1.15 cm and multiple tumors as independent risk factors, which was confirmed by the AUC value of 0.699 assigned to these indications. In patients whose iconography or pathology suggests suspicious lateral lymph node metastasis accompanied by the above risk factors, a more aggressive surgical approach to lateral lymphadenectomy is necessary. However, since such a surgical approach requires surgeons to have much experience and skill and because of the potential effects of surgery on regional function and complications, prophylactic LND should be chosen with prudence. This is especially advised in those patients who do not have risk factors or for whom clinical evidence does not suggest such a surgical approach.

This study has several potential limitations. First, it is a retrospective, single-institution study, which inherently carries the limitations of a nonrandomized design. Consequently, additional prospective and multicenter data are required to enhance the robustness of the results. Second, this study lacks long-term monitoring and research on the prognosis of lymph node metastasis. While our nomogram can identify patients at high risk for lymph node metastasis, it remains uncertain whether lymph node dissection will improve long-term survival. To address this gap, we are undertaking a comprehensive investigation into the prognosis of lymph node metastasis. Third, the majority of cases in this study involved thyroid microcarcinomas; thus, future research should aim to include a greater number of patients with papillary thyroid carcinoma (PTC) greater than 1 cm. Additionally, conducting risk analyses for genetic testing is currently unfeasible due to the high costs associated with genetic testing.

While our study provides valuable insights into the risk factors associated with lymph node metastasis in papillary thyroid cancer (PTC), it is important to acknowledge that the patient cohort is drawn from a single institution. As such, the applicability of our findings to broader or more diverse patient populations may be limited. Regional variations in healthcare practices, differences in surgical techniques, genetic predispositions, and environmental factors could influence the outcomes observed in different populations. For example, certain populations may exhibit distinct tumor biology or varying rates of lymph node metastasis due to regional or genetic factors, which were not accounted for in our cohort.To improve the generalizability of these findings, future research should consider expanding the study across multiple centers, including both national and international institutions. This would allow for the collection of a more diverse dataset, capturing variations in patient demographics, clinical practices, and healthcare resources. Multicenter or multinational studies would also help validate our predictive models and risk factors across different healthcare settings, ensuring that the recommendations provided are applicable to a wider range of PTC patients globally.

Moreover, further studies with larger sample sizes would help strengthen the statistical power of the analysis and enable the identification of additional risk factors that may be unique to specific subgroups of patients. Such efforts are crucial for refining treatment guidelines and improving the personalization of surgical interventions for patients with PTC.

## 5 Conclusions

The rate of metastasis is high among PTC patients even at the early stage, and the risk factors for LNM are different from those for CNM. However, while central neck lymph nodes may be routinely dissected, LND should be treated with greater caution. To reduce the possibility of a second surgery and improve prognosis, under the precondition of regular CND, patients with tumors with a maximum diameter greater than 1.15 cm, especially those with multiple tumors, CNM and capsular invasion, should be subjected to aggressive lateral lymphadenectomy. The extent of lymph node dissection should be carefully decided with reference to risk factors to eradicate the tumor with minimal damage to patients.

## Data Availability

The original contributions presented in the study are included in the article/supplementary material. Further inquiries can be directed to the corresponding author.
